# Thyroid Hormone Promotes Remodeling of Coronary Resistance Vessels

**DOI:** 10.1371/journal.pone.0025054

**Published:** 2011-09-22

**Authors:** Olga V. Savinova, Yingheng Liu, Garth A. Aasen, Kai Mao, Nathan Y. Weltman, Brett L. Nedich, Qiangrong Liang, A. Martin Gerdes

**Affiliations:** Cardiovascular Health Research Center, Sanford Research/University of South Dakota, Sioux Falls, South Dakota, United States of America; Brigham and Women's Hospital, United States of America

## Abstract

Low **thyroid hormone (TH)** function has been linked to impaired coronary blood flow, reduced density of small arterioles, and heart failure. Nonetheless, little is known about the mechanisms by which THs regulate coronary microvascular remodeling. The current study examined the initial cellular events associated with coronary remodeling induced by **triiodothyronine (T3)** in hypothyroid rats. Rats with established hypothyroidism, eight weeks after surgical **thyroidectomy (TX)**, were treated with T3 for 36 or 72 hours. The early effects of T3 treatment on coronary microvasculature were examined morphometrically. Gene expression changes in the heart were assessed by quantitative PCR Array. Hypothyroidism resulted in arteriolar atrophy in the left ventricle. T3 treatment rapidly induced small arteriolar muscularization and, within 72 hours, restored arteriolar density to control levels. Total length of the capillary network was not affected by TX or T3 treatment. T3 treatment resulted in the coordinate regulation of Angiopoietin 1 and 2 expression. The response of Angiopoietins was consistent with vessel enlargement. In addition to the well known effects of THs on vasoreactivity, these results suggest that THs may affect function of small resistance arteries by phenotypic remodeling of **vascular smooth muscle cells (VSMC)**.

## Introduction

THs affect diastolic and systolic function and peripheral vascular tone [1]. Chronic hypothyroidism can lead to severe left ventricular dysfunction, chamber dilatation, impaired coronary blood flow, reduced density of small arterioles, and eventual heart failure [2]. Many clinical studies also suggest that borderline low thyroid conditions have a negative prognostic value in cardiac patients. Not only do heart failure patients have a higher incidence of low TH function, the HUNT study showed that higher levels of **thyroid stimulating hormone (TSH)**, which are still within the normal range, are associated with increased coronary artery disease mortality in women and unfavorable serum lipids in all patients [3], [4]. The topic of low thyroid function in heart failure and potential benefits of treatment was reviewed recently by Gerdes and Iervasi [5].

Animal studies demonstrated beneficial effects of THs on cardiac remodeling and function in dilated cardiomyopathy, hypertension, and myocardial infarction without increasing heart rate above normal values [6], [7], [8], [9]. Of particular relevance to the current study, we demonstrated impaired resting and maximum blood flow in BIO-TO2 hamsters, subclinical hypothyroidism, and normalization of blood flow after TH treatment, which led to dramatic benefits on underlying cardiac pathology [6]. Cumulatively, the above reports suggest that more information is needed regarding TH regulation of small resistance vessels in the heart since they may play an important role in coronary blood flow in heart diseases, particularly in borderline low TH conditions.

The current study examines rapid coronary vascular remodeling in hypothyroid rats after treatment with T3. While the vasomotor effects of THs have long been recognized, results from the current study suggest that THs may work through another previously unrecognized vascular remodeling mechanism to alter coronary blood flow. This TH-dependent mechanism may be particularly relevant in borderline low TH conditions, which are common in heart failure.

## Materials and Methods

### Animal model and study design

The use of animals in this study conformed to the Public Health Service Guide for Care and Use of Laboratory Animals. Experiments were reviewed and approved by the Sanford Research/University of South Dakota Institutional Animal Care and Use Committee (Approval ID 08-11-08-11C). Surgical services were provided by Charles River Laboratories (Wilmington, MA). Female Sprague-Dawley rats had TX at the age of 10 weeks. TX rats were aged a minimum of 8 weeks to establish hypothyroidism then randomly divided into three experimental groups (12 rats/group). To ensure adequate TX surgery and establishment of hypothyroidism, rats with normal heart rate, body temperature, and TSH levels were excluded from the study. Two of three groups were treated with T3 (14 µg/kg/day, every 24 hours) for 36 h and 72 h, respectively. The other group was treated with sterile saline as placebo. Twelve age- and sex-matched Sprague-Dawley rats without surgery served as controls. All animals were exposed to a 12-hour light-dark cycle and given standard rat chow and water *ad libitum*. Serum concentration of TSH were determined using a TSH ELISA kit (Calbiotech, Spring Valley, CA). Serum T3 and **Thyroxine (T4)** were measured using Total T3 and Total T4 ELISA kits, respectively (Alpha Diagnostics, San Antonio, TX). Body weight, heart weight, and heart rate (via echocardiogram) were recorded during terminal experiments. After echocardiograms were collected, each animal was deeply anesthetized with 5% isofluorane and the chest cavity opened. Hearts were arrested in diastole and vasorelaxation was achieved by injecting freshly prepared 0.2% 2,3-butanedione monoxime and 0.1% adenosine solution into the **left ventricle (LV)**. Hearts were then quickly removed, rinsed in ice-cold phosphate buffered saline (PBS), blotted and weighed. LVs were dissected and weighed. Apical and basal slices of LVs were stored frozen for RNA and protein analysis. The remaining tissue was sliced transversely, embedded in OCT compound (Sakura Finetek Inc, Torrance, CA) and frozen, or immersion fixed in 10% formalin for morphometric analysis.

### Morphometry

All fluorescence images were acquired using an Olympus FluoView 1000 confocal laser scanning microscope (Olympus Corp., Tokyo, Japan). Changes in myocardial arteriolar density were quantified morphometrically as reported previously [10]. In addition, we modified the original protocol to enhance recognition of the smallest arterioles. Combined **Isolectin B4 (IB4**, marker of **endothelial cells, EC**s) and **α smooth muscle actin (α-SMA**, immunohistochemical marker of VSMC) were used instead of α-SMA only. This enabled consistent identification of the smallest arterioles, which often had incomplete smooth muscle cell coverage. 3D reconstruction of these small vessels clearly identified them as arteriolar in nature. In previous studies, these vessels were not counted. De-paraffinized, re-hydrated tissue sections were heated to 95°C in 10 mM Na Citrate, pH 5.0 containing 0.05% Tween-20 for 20 minutes, cooled to room temperature, and incubated with blocking solution for 30 minutes. Blocking and antibody diluent solution consisted of 2% Bovine serum albumin in Tris-buffered saline containing 0.05% Tween-20 and 1 mM CaCl_2_. Sections were stained with **fluorescein isothiocyanate-conjugated IB4 (IB4-FITC**; Vector Labs, Burlingame, CA; final dilution 1∶200) and **α-SMA antibody labeled with Cy3 fluorescent dye (α-SMA-Cy3**; Sigma, St. Louis, MO; final dilution 1∶5000) for one hour, rinsed in antibody diluent solution and coverslipped with Fluoromount G (EMS, Hatfield, PA). Myocardial arterioles were visualized by confocal microscopy at 20× magnification (35 random fields/heart). Arteriolar **length density (LD**, average length of arterioles/unit myocyte volume [11]) was calculated based on the following formula: LD (mm/mm^3^) = ∑ (a/b)/M, where a and b are the maximum and minimum external arteriolar diameters, respectively, and M is the tissue area. Arteriolar LD was converted to total arteriolar length by multiplying LD (mm/mm^3^) by LV weight expressed in mm^3^ (∼1 mg of cardiac tissue is equivalent to 1 mm^3^).

Capillary density was determined by confocal microscopy of methanol fixed cryosections of OCT embedded LV tissue from areas containing only circular, cross-sectioned capillary profiles stained with IB4-FITC and α-SMA-Cy3 (mean sampling area ∼0.4 mm^2^/per heart). The number of IB4 positive (and α-SMA negative) capillaries was normalized to tissue area and converted to total capillary length by multiplying numerical capillary density (number/mm^2^) by LV weight expressed in mm^3^.

Ki67-specific antibody (Abcam, Cambridge, MA, final dilution 1∶100) and **4′,6-diamidino-2-phenylindole (DAPI**, Molecular Probes, Eugene, OR) were used to detect proliferating nuclei in 5 µm cryosections. Secondary antibodies conjugated to AlexaFlour-488 or AlexaFlour-568 (Molecular Probes, Eugene, OR) were diluted 1∶1000. IB4-FITC was used to detect ECs. VSMCs were detected with α-SMA-Cy3. Pericytes were detected with antibody specific to NG2 or PDGFR-β (gift from Dr. William Stallcup, Sanford-Burnham Institute for Medical Research, La Jolla, CA). Co-localization of labeling was determined using confocal microscopy. 100 microscopic fields per sample (40×) were examined.

### Echocardiography

Heart rate was determined echocardiographically using a VisualSonics Vevo 660 high-resolution imaging system with a 25-MHz RMV-710 transducer (Toronto, ON, Canada) as reported by our group previously [10]. Animals were lightly anesthetized with 1.5% isofluorane and M-mode images were obtained from the short axis of the LV at the level of the papillary muscles.

### Quantitative PCR array

RNA was isolated from 50–100 mg of cardiac tissues using TRIzol reagent followed by RNA purification with the PureLink RNA Mini kit and DNA digestion using the PureLink DNase kit (Invitrogen, Carlsbad, CA). Equal amounts of high quality RNA from each sample within a particular experimental group were pooled. cDNA was synthesized using RT^2^ First Strand kit (SABiosciences, Frederick, MD). Expression of 84 angiogenesis inhibitors and agonists was analyzed by quantitative PCR using the RT^2^ Profiler qPCR Array (cat# PARN-072; SABiosciences, Frederick, MD) per manufacturer's instructions. ABI 7500 real-time PCR instrument operated by Software Version 1.3.0 was used for all experiments (Life Technologies, Carlsbad, California). Expression data were analyzed using SABiosciences expression analysis template in Excel (Microsoft Office 2008).

### Statistical analyses

All values are presented as means ± SEM. One-way ANOVA models were used for all responses. Post hoc Dunnett's test was used to compare all treatment groups with the control group and with TX group. Statistical analyses were performed using SigmaStat version 3.5 (Aspire Software International, Ashburn, VA) and significance was accepted at p<0.05. Linear regression analysis was performed using SigmaStat version 3.5.

## Results

### Physical data

**Figure 1:**
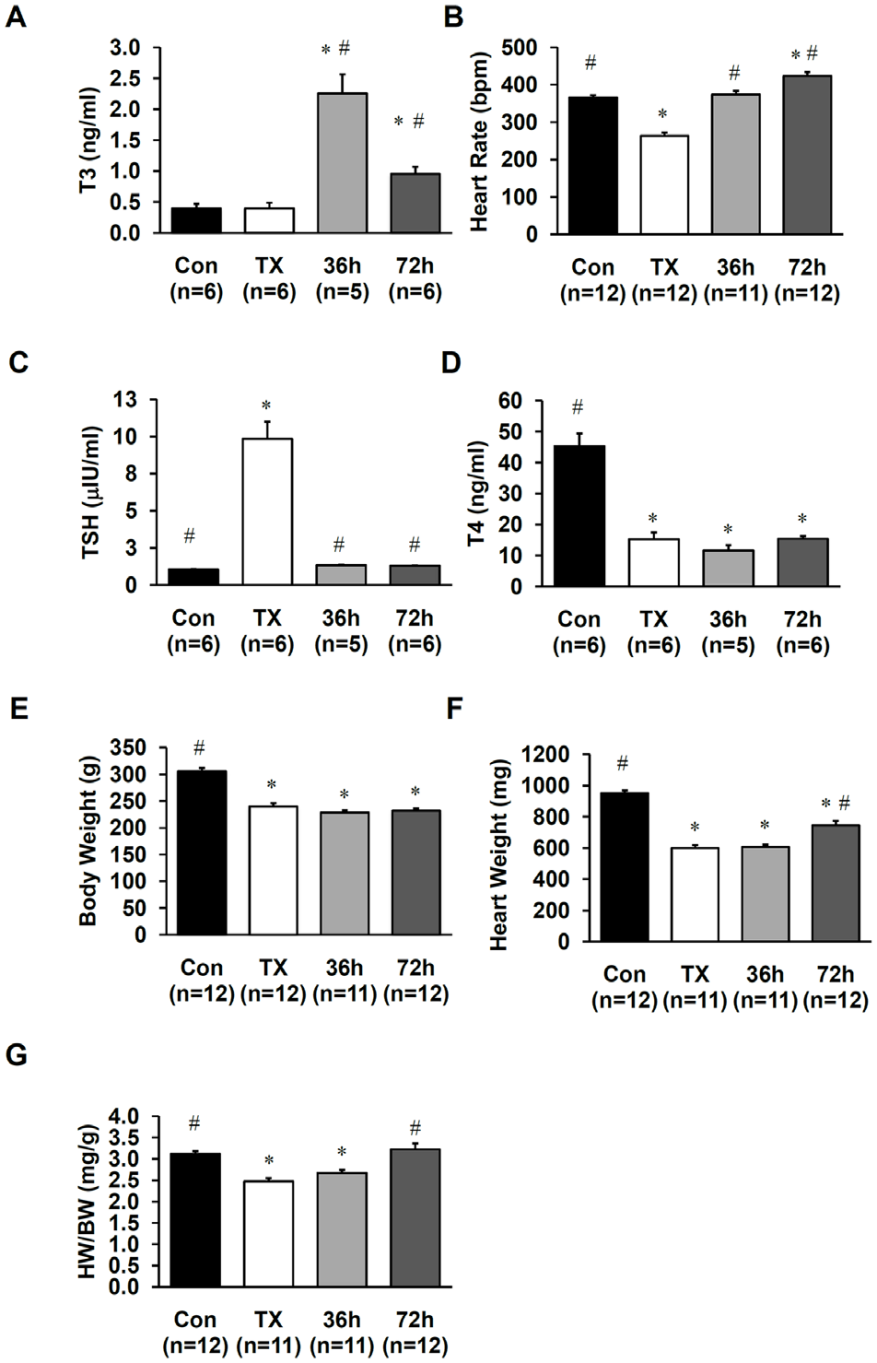
Experimental model and response to treatment. A–G. Changes in serum T3 (A), heart rate (B), serum TSH (C), serum T4 (D), body weight (E), heart weight (F), and heart weight body weight ratio (HW/BW, G). Con, control; TX, thyroidectomized rats; 36 h, TX rats treated with T3 (14 µg/kg/day) for 36 h; 72 h, TX rats treated with T3 for 72 h; bpm, beats per minute. Values are means ± SEM. * p<0.05 vs. control; # p<0.05 vs. TX.

Rats with established hypothyroidism (eight weeks after TX) received intraperitoneal injections of T3 (14 µg/kg) every 24 hours. One group of TX rats was treated for 36 hours (they received a total of two injections) and another group was treated for 72 hours (this group received three injections.). The dose, 14 µg/kg/day, was selected to produce a pronounced pharmacological effect of T3 on the heart. T3 treatment resulted in elevated levels of circulating T3 (Figure 1A). Heart rate was lower in TX rats compared to control rats and was normalized to control levels after 36 hours of T3 treatment. 72 hours of T3 treatment increased heart rate above the control levels (Figure 1B). Serum TSH was higher in TX rats when compared to control and was reduced to a normal level after 36 hours of T3 treatment (Figure 1C). Circulating T4 concentration in TX animals was abnormally low and was not affected by T3 treatment (Figure 1D). TX rats showed significant reduction of body weight, heart weight, and displayed cardiac atrophy (reduced **heart weight to body weight ratio, HW/BW**) compared to controls (Figure 1E–G). T3 treatment for 72 hours restored HW/BW ratio to control levels (Figure 1G).

### Myocardial capillaries

**Figure 2:**
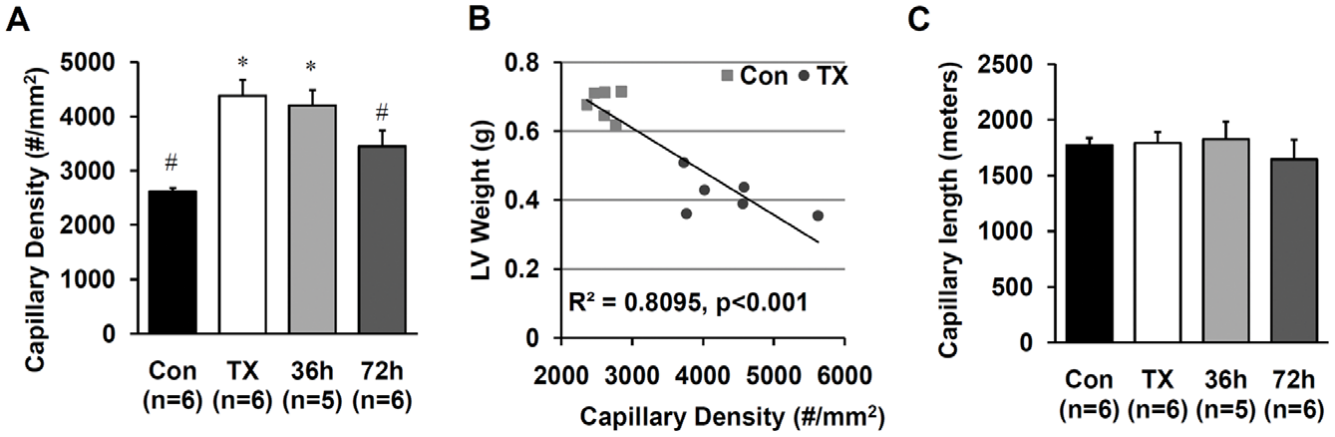
Capillaries are preserved in the hearts of hypothyroid rats. A. Capillary density; B. Linear regression analysis of the correlation between capillary density and LV weight of the control and TX groups combined; C. LV capillary length. Values are means ± SEM. * p<0.05 vs. control; # p<0.05 vs. TX.

An increase in capillary density in TX animals (which displayed significant cardiac atrophy) and reduction of capillary density in T3-treated animals was observed (Figure 2A). Numeric capillary density inversely correlated with LV weight in TX and T3-treated animals (Figure 2B). This observation suggests a passive change in intercapillary distance due to cardiomyocyte atrophy (in TX animals) or growth (in T3 treated animals). Indeed, when total capillary length per LV was calculated, no changes were observed between the groups (Figure 2C).

### Myocardial arterioles

**Figure 3:**
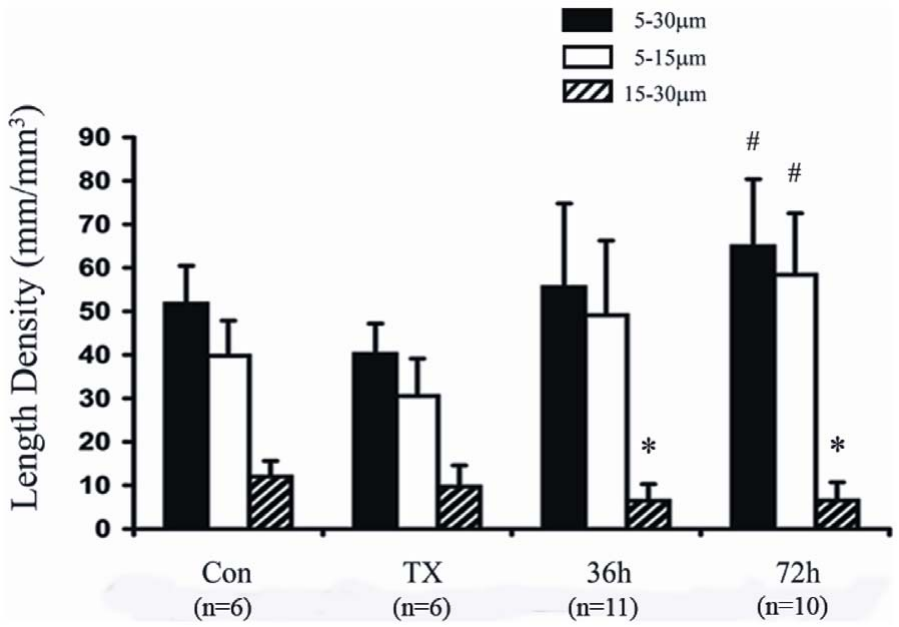
Arteriolar length density in adult myocardium. Values are means ± SD. * P<0.05 vs. control; # P<0.05 vs. TX.

Changes in arterioles are shown in Figure 3. While arteriolar LD was reduced by 23% in the 5 to 30 µm size range in TX rats versus controls, this did not quite reach statistical significance as reported in our two previous studies of hypothyroid rats [10], [12]. Compared to TX rats, LD of the 5 to 30 µm arterioles increased by 61% at 36 hours and 91% at 72 hours.

Since the rapid T3-induced increase in small arterioles could be due to vessel splitting (intussusception), extensive examination was made of vessels in confocal image stacks. No evidence of arteriolar splitting was ever observed.

**Figure 4:**
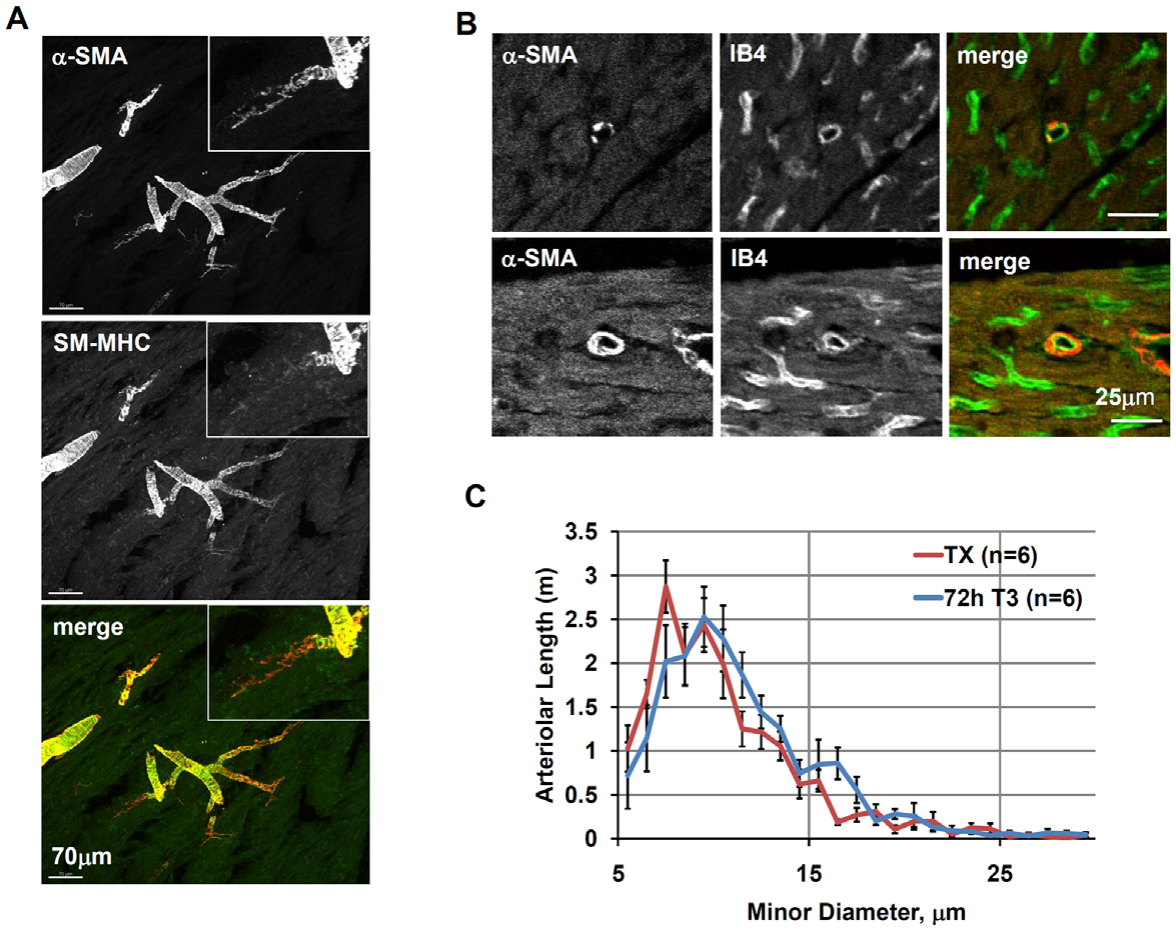
Induction of muscularization of small arterioles by T3 treatment of TX rats. A. Confocal stack projection images of representative arteriolar branch point in thick (30 µm) cryosections; B. Modified morphometric protocol increases the sensitivity of measurement of small arterioles with incomplete VSMC coverage; examples of smaller (top panels) and larger vessels (bottom panels); C. Changes of arteriolar size distribution 72 h after initiation of T3 treatment as compared to arteriolar size distribution in TX rats; α-SMA – alpha smooth muscle actin, IB4 – isolectin B4, SM-MHC – smooth muscle myosin heavy chain. Values are means ± SEM.

It has been shown that newly formed arterioles are reactive with α-SMA antibody but not with **smooth muscle myosin heavy chain (SM-MHC)** antibody [13]. As these arterioles mature, they start to express SM-MHC [13]. Consequently, potential T3-related differences in the expression pattern of α-SMA and SM-MHC were examined in an effort to detect arteriolar sprouting. Larger arterioles labeled clearly with both markers (Figure 4A). Although a dramatic reduction in SM-MHC labeling intensity of the smallest arterioles was observed, there were no group differences.

Extensive confocal observations of tissue sections co-labeled with IB4 and α-SMA revealed that many of the smallest arterioles with a discontinuous layer of smooth muscle were being excluded with the single labeling approach due to uncertain classification. Consequently, arteriolar length was reassessed using this dual labeling method. It should be noted that this new approach led to improvement in objectivity and reproducibility in measuring small arterioles. Figure 4B demonstrates how easily small arterioles with a discontinuous or continuous smooth muscle cell layer can be identified using dual labeling. In TX rats treated with T3 for 72 hours, an increase in small arterioles (10–18 µm diameter) was observed (Figure 4C). Of particular note, T3 treatment also led to a reduction in total length of the smallest arterioles (5–9 µm range), which included arterioles with a discontinuous smooth muscle layer (Figure 4C).

### T3 induction of cell proliferation

**Figure 5:**
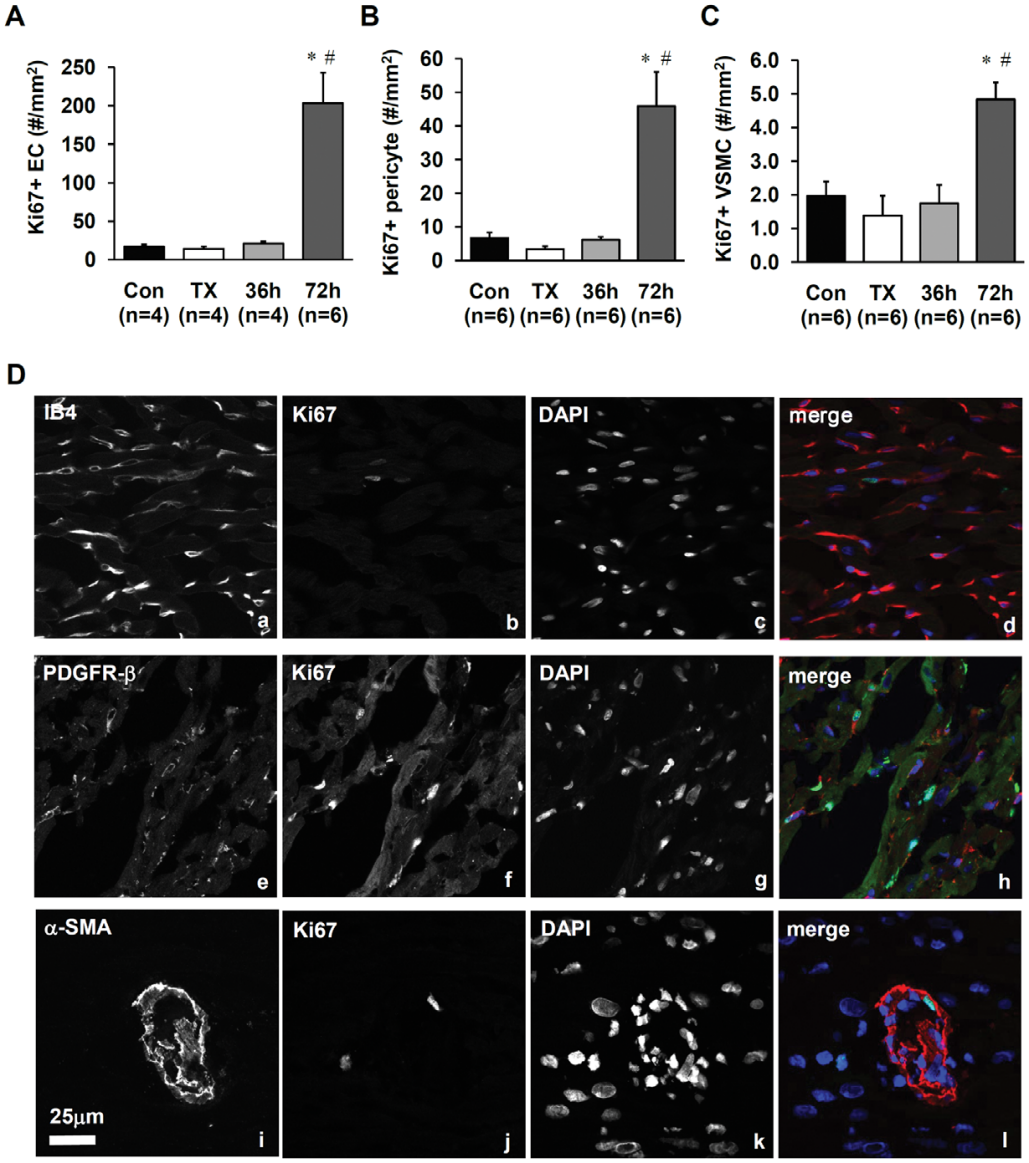
T3 induces proliferation of microvascular cells in the left ventricle. A–C. Quantitation of Ki67-positive EC (A), pericytes (B), and VSMC (C); D. Representative images of Ki67-positive nuclei of EC (a–d; IB4^+^), pericytes (e–h; PDGFR-β^+^) and VSMC (i–l; α-SMA^+^) in the left ventricle (100×). Values are means ± SEM; * p<0.05 vs. control, # p<0.05 vs. TX.

Proliferating cells were detected by the presence of Ki67 antigen in nuclei, a cellular marker for proliferation expressed during all active phases of the cell cycle (G1, S, G2, and mitosis). There were no changes in cell proliferation 36 hours after T3 injection (Figure 5). T3 treatment for 72 hours, however, led to a significant increase in the number of Ki67-positive EC, VSMCs, and pericytes. c-Kit labeling was examined to study the possible role of stem cells in this process. Labeling frequency was extremely low (5–9 positive cells per transverse slice of LV and septum of rat heart, data not shown) and there were no differences between groups indicating that stem cells may not play a role in the early stage of T3 mediated arteriolar remodeling induced in hypothyroid animals.

### T3-induced gene expression

To investigate molecular mechanisms involved in T3-dependent arteriogenesis, a qPCR array approach targeting 84 potential activators and inhibitors of microvascular growth was used. We suspected that, regardless of the etiology of hypothyroidism, TH treatment of hypothyroid animals should elicit similar gene expression changes. To test our hypothesis we used two different models of hypothyroidism: a thyroidectomy model (TX) and **propylthiouracil (PTU**) induced model of hypothyroidism (0.025% PTU was delivered in drinking water over the course of six weeks; archived frozen LV tissue samples were used to comprise PTU experimental groups). Variation of expression of qPCR Array genes was examined across experimental groups representing different levels of TH function: euthyroid control (n = 3), hypothyroid (TX or PTU; n = 4) and reconstituted hypothyroid (TX+T3 or PTU+T3, n = 4). Each sample within an experimental group consisted of a pool of RNA (4–5 animals per RNA sample). Expression was normalized to the expression of housekeeping genes, Rplp1 and Ldha, which deviated minimally across all experimental groups.

Hypothyroidism significantly affected the expression of five genes: expression of **Angiopoietin 1 (Angpt1)** was downregulated and the expression or the remaining four genes was upregulated (p<0.05, Table 1). Notably, expression pattern of four out of five genes affected by hypothyroidism (Angpt1, CD55, Cited1, and Mdk) was reversed by T3 treatment of hypothyroid animals, (p<0.05, Table 1). Remarkably, T3 treatment upregulated expression of Angpt1 and downregulated expression of **Angiopoietin 2 (Angpt2**, p<0.05, Table 1). This observation was interesting because these two molecules might serve as mutual antagonists in cardiac angiogenesis. Overall, the qPCR array experiment identified the Angiopoietin signaling system as one of the likely targets of TH in the heart.

**Table 1:** Thyroid hormone-dependent cardiac mRNA transcripts.

Gene Symbol	Gene Name	Fold Regulation	p-value
**Genes Over-Expressed in Hypothyroid vs. Control Rats**			
Cd55	CD55 molecule, decay accelerating factor for complement	2.5	0.001
Cited1	Cbp/p300-interacting transactivator with Glu/Asp-rich carboxy-terminal domain 1	1.9	0.035
Cxcl10	chemokine (C-X-C motif) ligand 10	2.6	0.027
Mdk	midkine	7	0.01
**Genes Under-Expressed in Hypothyroid vs. Control Rats**			
Angpt1	angiopoietin 1	−2.3	0.047
**Genes Over-Expressed in T3-treated vs. Untreated Hypothyroid Rats**			
Angpt1	angiopoietin 1	2.6	0.021
**Genes Under-Expressed in T3-treated vs. Untreated Hypothyroid Rats**			
Angpt2	angiopoietin 2	−1.7	0.046
Angptl3	angiopoietin-like 3	−4.2	0.044
Cd55	CD55 molecule, decay accelerating factor for complement	−2	0.005
Cited1	Cbp/p300-interacting transactivator with Glu/Asp-rich carboxy-terminal domain 1	−4.8	0.001
Erbb2	v-erb-b2 erythroblastic leukemia viral oncogene homolog 2	−3.1	0.027
Foxo4	forkhead box O4	−2.3	0.024
Mdk	midkine	−7.1	0.003
Smo	smoothened homolog (Drosophila)	−2.4	0.031

## Discussion

This study investigated early cellular changes triggered by TH in the coronary microvasculature of hypothyroid rats. Thyroid hormones and their analogues are known to be pro-angiogenic in adult heart and can stimulate arteriolar growth in normal heart as well as after myocardial infarction [14], [15], [16], [17], [18]. Previous studies in our lab have shown that hypothyroidism induced by PTU treatment led to impaired myocardial blood flow and a significant reduction in myocardial arterioles [2] as early as three weeks after initiating the PTU treatment [19]. These changes have also been confirmed after inducing hypothyroidism by thyroidectomy (TX) in rats. In the TX model of hypothyroidism, microvascular impairment was prevented by thyroxine (T4) [10] or 3,5-diiodothyropropionic acid (DITPA) [12]. The current study demonstrated that arteriolar length in the TX model was restored within 72 hours after initiation of T3 treatment. To determine the cellular mechanism responsible for restoration of the microvasculature in the heart, early morphological and cellular events were investigated.

### Animal model and treatment dosage selection

We and others have shown previously that T3 treatment induces arteriolar growth in the heart. The mechanism of T3 action on arterioles, however, was not clear. To investigate the mechanism of T3 action on arterioles, we choose an experimental system which can maximize the growth response. We compared animals across three different phenotypes: normal, hypothyroid, and hypothyroid animals treated with T3. The goal of the study was to detect changes consistent with the thyroid state across different levels of thyroid function. The model of hypothyroidism was effective because, despite the apparent lack of suppression of circulating T3, thyroidectomized animals had elevated TSH levels, reduced heart rate, and reduced HW/BW ratio, clear signs of hypothyroidism (Figure 1). The levels of circulating T3 in both control and TX groups were at the lower end of the detection limit. The method of T3 detection used in this study (human total T3 ELISA kit) is optimized for the detection of hyperthyroidism, with less sensitivity at the lower end where circulating T3 in TX and control rats were primarily grouped. We chose to treat hypothyroid animals with 14 µg/kg/day T3 because previous work from Morreale de Escobar's laboratory [20] and from our studies [10] indicated that circulating levels of T3 may not accurately reflect cardiac tissue level of T3 upon hormone replacement. Moreover, our ongoing studies indicate that the T3 dose necessary to restore cardiac function is at least three times higher (∼10 µg/kg/day, over the course of two weeks) than the T3 dose necessary to restore circulating levels of T3 (AM Gerdes and NY Weltman, unpublished observations). Thus, we selected a pharmacological dose leading to a slight elevation of heart rate to ensure a strong T3-angiogenic stimulus without inducing marked hyperthyroidism. We also chose to examine animals at early time periods (36 and 72 hours) since little is known about the early changes elicited by T3 on coronary vasculature both at the molecular and at the anatomical levels during this period.

### Arteriolar atrophy in hypothyroid rat heart

Arteriolar length density (mm/mm∧3) was not significantly different between control and TX rats in this study. When corrected to the reduced LV weight, however, total arteriolar length in hypothyroid rats was significantly reduced in TX rats compared to control rats (data not shown). Based on our cumulative results, we interpreted this reduction as arteriolar atrophy. The mechanisms that trigger coronary arteriolar atrophy in hypothyroidism are likely to be the same as those that cause left ventricular atrophy: decreased rate of metabolism and reduced blood flow. Of note, total capillary length in LV was not affected by thyroidectomy or T3 treatment (Figure 2). It appears that intercapillary distance in the vascular bed of the heart passively changes depending on TH-induced changes in myocyte cross-sectional area.

### T3 does not induce sprouting and splitting of coronary arterioles in TX rats

The observation that T3 induced significant arteriolar growth within only three days was remarkable (Figure 3). Could sprouting angiogenesis account for such a rapid increase in arterioles? To evaluate this possible mechanism, we performed extensive histological examination of cryosections in search of spatial patterns of cell proliferation suggestive of sprouting. Sprouts in whole tissue should be characterized by the presence of proliferating nuclei aligned in a linear direction with the vessel. After examining of hundreds of sections from T3 treated animals, we were unable to observe any examples of arteriolar or capillary sprouts. Moreover, no significant proliferation was detected at 36 hours of T3 treatment and only single proliferating ECs, pericytes, and VSMCs were observed interspersed within the tissue after 72 hours of treatment (Figure 5). Additionally, the labeling pattern for α-SMA and SM-MHC was similar in all animal groups with reduced SM-MHC labeling being a common feature of the smallest arterioles (Figure 4A). If sprouting angiogenesis of small arterioles had occurred, a T3-mediated increase in the number or length of SM-MHC positive small arterioles would have been anticipated as reported previously in young growing animals [13].

The presence of Ki67 positive cells at the 72 hour timepoint likely marks the beginning of capillary proliferation, which has been noted at later timepoints by others [21]. Regarding the rapid T3-mediated increase in arteriolar density, it is safe to conclude that sprouting angiogenesis did not play a role in the time frame examined.

The potential role of intussusception (vessel splitting) was examined next. Intussusception can occur very quickly with minimal cell proliferation and has been observed in fetal and early postnatal heart [22]. After extensive examination of IB4/α-SMA labeled arterioles by confocal microscopy, we were unable to find a single example of intussusception. Thus, morphogenesis of coronary arterioles by splitting is unlikely to play a role in the model and time frame examined.

### T3 induces muscularization of VSMC

When quantitating arterioles with an α-SMA label only, many small individual positive cells were noted. These cells were routinely ignored in arteriolar quantitation in previous studies because they are not associated with a continuous vascular smooth muscle cell layer. Spatial examination of vessels after dual labeling with IB4 (EC marker) and α-SMA (VSMC marker) revealed that these α-SMA positive cells represented a subset of smaller arterioles with discontinuous α-SMA labeling (Figure 4B). This arteriolar subset (5–9 µm diameter) was decreased in T3-treated animals compared to untreated hypothyroid rats (Figure 4C). Thus, it appears that T3 promotes a more differentiated phenotype of VSMCs characterized by re-expression of α-SMA. VSMCs are known to have a high capacity for phenotypic “switching”. Phenotypic switching involves loss of expression of α-SMA and other markers of differentiated VSMCs and re-expression of factors that suppress VSMC differentiation, including Krüppel-like zinc finger 4 (Klf4), HERP, and Elk [23].

### Thyroid function correlates with the expression of angiopoietins 1 and 2 in rat heart

It has been demonstrated that TH binds to surface receptors on ECs and activates intracellular signaling pathways promoting sprouting angiogenesis [24], [25]. Less is known about the action of TH on VSMCs. To identify molecular factors that can play a role in T3-induced coronary remodeling, we used a commercially available qPCR array targeting expression of 84 inducers and inhibitors of angiogenesis (Table 1). We looked for significant changes in gene expression induced by hypothyroidism and restored by T3 treatment in adult rat heart. We observed that expression of Angpt1 in heart positively correlated with TH function. Conversely, the expression of Angpt2 (a negative regulator of Angpt1 [26]) was decreased by T3 treatment. In neonatal development, Angpt1 acts to enlarge blood vessels by recruiting periendothelial support cells without inducing sprouting [27], [28], [29]. In adult organisms, Angpt1 gene transfer could modulate collateral vessel development (as has been shown in a rabbit model of hind limb ischemia) [30]. It appears that TH could regulate the angiopoietin system in adult heart. Further studies are needed to delineate the mechanisms of action of angiopoietins in coronary remodeling.

Angpt1 was previous characterized as a pro-angiogenic target of the TH analogue DITPA [16]. The genomic sequence upstream of *angpt1* gene in rat and human, indeed, contains two conserved TH response elements. It would be interesting to examine in a future study if TH receptor directly binds to the promoter of Angpt1 in VSMSs and whether ligation of TH receptor influences the levels of Angp1 gene expression.

Chronic bradycardia was shown to have a beneficial effect on coronary revascularization in rats with myocardial infarction by upregulation of several growth factors and their receptors, namely, VEGF, Flt-1, and bFGF. In contrast, the angiopoietin system was not affected by bradycardia [31]. Thyroid hormones appear to influence coronary remodeling by different mechanisms, with expression of Angpt1 and Angpt2 in the heart prominently affected. It is possible, that despite increasing heart rate, TH deliver stimuli sufficient to sustain beneficial remodeling of resistance vessels to improve coronary circulation.

Cumulatively, our observations suggest that thyroid hormones may play an important role in promoting a more differentiated phenotype of VSMCs. It appears that the “missing” small arterioles in hearts from hypothyroid rats observed by our group previously may actually be present in a dedifferentiated state. Since we have previously shown that this alteration in small arterioles is associated with a dramatic reduction in maximum coronary blood flow in hypothyroid rats [2], T3-induced modulation of VSMC phenotype may play a major role in the regulation of blood flow. Increased muscularization of small arterioles in response to T3 defines an anatomical feature that might support better regulation of coronary flow reserve. Of note here, BIO-TO2 hamsters have subclinical hypothyroidism and a pattern of impaired coronary blood flow similar to that of hypothyroid rats [6]. Treatment of BIO-TO2 hamsters with thyroid hormones restored coronary blood flow to normal [6]. Although cardiac patients are typically classified as ischemic or non-ischemic based on patency of major coronary arteries, evidence from patients with idiopathic dilated cardiomyopathy has demonstrated the presence of significant vascular dysfunction [32], [33], [34]. When considering the high incidence of low thyroid function in heart failure patients, it is likely that impaired blood flow and flow reserve in many of these patients may be related to the effects of thyroid hormones on small coronary resistance vessels, as observed here.
